# Presentation of SLE after COVID vaccination in a pediatric patient

**DOI:** 10.1186/s41927-022-00313-8

**Published:** 2022-12-20

**Authors:** Meghan Corrigan Nelson, Heather Rytting, Larry A. Greenbaum, Baruch Goldberg

**Affiliations:** 1 Division of Pediatric Rheumatology, Department of Pediatrics, Emory University School of Medicine, Atlanta, GA USA; 2 Children’s Healthcare of Atlanta, Atlanta, USA; 3 Division of Pediatric Nephrology, Department of Pediatrics, Emory University School of Medicine, Atlanta, GA USA; 4 Division of Pathology, Children’s Healthcare of Atlanta, Atlanta, USA

**Keywords:** Systemic lupus erythematosus, COVID-19, Vaccination, SARS-CoV2, Lupus nephritis, Case report

## Abstract

**Background:**

The outbreak of severe acute respiratory syndrome coronavirus 2 has had an enormous impact on global health. Vaccination remains one of the most effective interventions for disease prevention. Clinically significant vaccine side effects are uncommon, though autoimmune-mediated disease occurs in a small percentage of vaccine recipients. Systemic lupus erythematosus (SLE) is a multisystem autoimmune disease that is associated with significant morbidity and mortality. Childhood-onset SLE tends to have more severe disease manifestations than adult-onset SLE. In adults, there are a few reported cases of SLE developing soon after coronavirus disease 2019 (COVID-19) mRNA vaccination.

**Case presentation:**

A 14-year-old previously healthy male developed laboratory and clinical evidence of SLE, including maculopapular malar rash, arthritis, pleuritic chest pain, and class V (membranous) lupus nephritis, 2 days after his third dose of the Pfizer-BioNTech COVID-19 vaccine. The patient’s symptoms improved after initiation of prednisone and mycophenolate mofetil. We also summarize eleven prior case reports describing SLE after COVID-19 vaccine in adults.

**Conclusion:**

To our knowledge, this is the first reported pediatric patient with new onset SLE following COVID-19 mRNA vaccination. While potential mechanistic links exist between COVID-19 vaccination and SLE development, additional studies are necessary to elucidate the exact nature of this relationship.

## Background

Systemic lupus erythematosus (SLE) is an autoimmune disease with multiple manifestations that most often presents in the second and third decades, and is much more common in females [1, 2]. Childhood onset SLE (cSLE) is more likely to cause kidney and neuropsychiatric disease, and has overall increased disease activity. Vaccinations have been proposed as potential triggers for the onset of SLE given their role in antigen stimulation, although these associations have not been confirmed in epidemiologic studies [1–5].

Severe acute respiratory syndrome coronavirus 2 (SARS-CoV-2) has caused 6.2 million deaths worldwide as of May 2022 [6]. Literature suggests SLE patients may be at an increased risk of poor outcomes with coronavirus disease 2019 (COVID-19) and vaccination is encouraged in SLE patients, especially those receiving potent immunosuppressive therapy [2, 7]. However, recent literature has shown that SLE patients exhibit more vaccine reactogenicity, with more frequent reports of fever, vomiting, and injection site redness following the SARS-CoV-2 mRNA Pfizer- BioNTech vaccine [8]. Furthermore, there are a number of case reports in adults describing SLE presenting after COVID-19 vaccination [7–12]. There are also reports of SLE exacerbations, including relapse of class V (membranous) lupus nephritis, after SARS-CoV2-vaccination in the adult population [12–15]. However, we could not identify any cases describing the development of cSLE or exacerbation of lupus nephritis during childhood. We report a pediatric patient who developed clinical symptoms of cSLE two days after administration of the 3rd dose of the SARS-CoV2 vaccination; he also had nephrotic-range proteinuria and a kidney biopsy demonstrated class V lupus nephritis.

## Case presentation

A fourteen-year-old Asian male (51 kg in weight) with no significant past medical history developed a non-photosensitive facial rash two days following his third dose of the SARS-CoV-2 mRNA Pfizer-BioNTech vaccine, and approximately eight months after his second vaccine. The patient was not receiving any medications and had not received any medications during the two months prior to the most recent vaccine. The rash quickly spread to his knees and arms; it was unresponsive to topical steroids and he was referred to dermatology. The dermatologist prescribed oral cephalexin given concern for secondary infection, but his rash did not improve. The patient subsequently developed bilateral arthralgias of his shoulders, hands, and knees. He also developed worsening hair loss, pleuritic chest pain, and photophobia. The patient had no relevant past medical history or family history of SLE or other autoimmune disorders. Additionally, he did not report any reactions with his previous COVID vaccines.

At his pediatrician visit three weeks after the vaccine, labs were notable for a positive antinuclear antibody (ANA 1:80 titer, nuclear speckled pattern) and positive autoantibodies against double-stranded deoxyribonucleic acid (dsDNA), Ro, Smith and ribonucleoprotein (RNP). He also had hypocomplementemia and an elevated erythrocyte sedimentation rate (ESR).

The patient was subsequently evaluated in pediatric rheumatology clinic five weeks after the COVID-19 vaccination. His blood pressure was 115/63, which is mildly elevated for age, sex and height (168.3 cm). His positive physical exam findings included arthritis of bilateral elbows, palatal erythema, maculopapular malar rash on face with flat, violaceous lesions on extremities. Patient also had capillary loop dilatation on nailfold capillaroscopy.

Laboratory evaluation at his initial rheumatology clinic visit included leucopenia (white blood cell count 3500 per µl), hemoglobin of 14.1 g/dL, platelet count of 140,000 per µl, hypoalbuminemia (albumin 2.6 g/dL), elevated ESR (126 mm/hour) and normal C-reactive protein. Repeat serology testing confirmed a positive ANA with a titer of 1:1280 (nuclear speckled pattern) and positive anti-dsDNA, anti-Smith, anti-RNP, and anti-Ro antibodies. The patient’s anti-histone antibody was also found to be high-titer positive (2.7 Units, reference < 1.0 negative). Lupus anticoagulant, anti-cardiolipin antibodies, beta-2-glycoprotein antibodies, and direct Coombs were negative. His immunoglobin G level was elevated at 1806 mg/dL (reference, 500–1590 mg/dL). His creatinine was 0.46 mg/dL and his urinalysis had 3 + protein and no red blood cells; the urine protein/urine creatinine ratio was 13.5 mg/mg (reference, < 0.2 mg/mg). His calculated glomerular filtration rate was 155 ml/min/1.73 m^2^ [16]. Chest X-ray as well as echocardiogram were normal. Table 1 summarizes the clinical findings, laboratory results, and management modalities in chronological order.

**Table 1:** Chronology of clinical features, laboratory results, and treatment modalities

	Day 0 (administration of 3rd SARS-CoV-2 Pfizer-BioNTech mRNA vaccine	Day + 4, saw pediatrician and was referred to dermatologist	Day + 20, seen by pediatric dermatology	Day + 35–38, seen by pediatric rheumatology and nephrology
Clinical features		Rash (face, with spread to knees and arms)	Persistent rash, joint pains, hair loss, and light sensitivity	Arthritis bilateral elbows, also found to have palatal erythema and persistent rash
Laboratory results		+ANA (1:80), +dsDNA, +Smith, +RNP, +Ro antibodies, low complements (C3 43, C4 7)		ANA 1:1280 with confirmed Smith/RNP, dsDNA, Ro+ antibodies; urine protein: creatinine elevated to 13.5 mg/mg
Treatment		Topical steroid cream	Oral cephalexin	Mycophenolate mofetil 1000 mg twice daily; hydroxychloroquine 300 mg daily; Prednisone 60 mg’ daily; famotidine 20 mg daily

*ANA* antinuclear antibody, *dsDNA* double-stranded deoxyribonucleic acid, *RNP* ribonucleoprotein

**Fig. 1:**
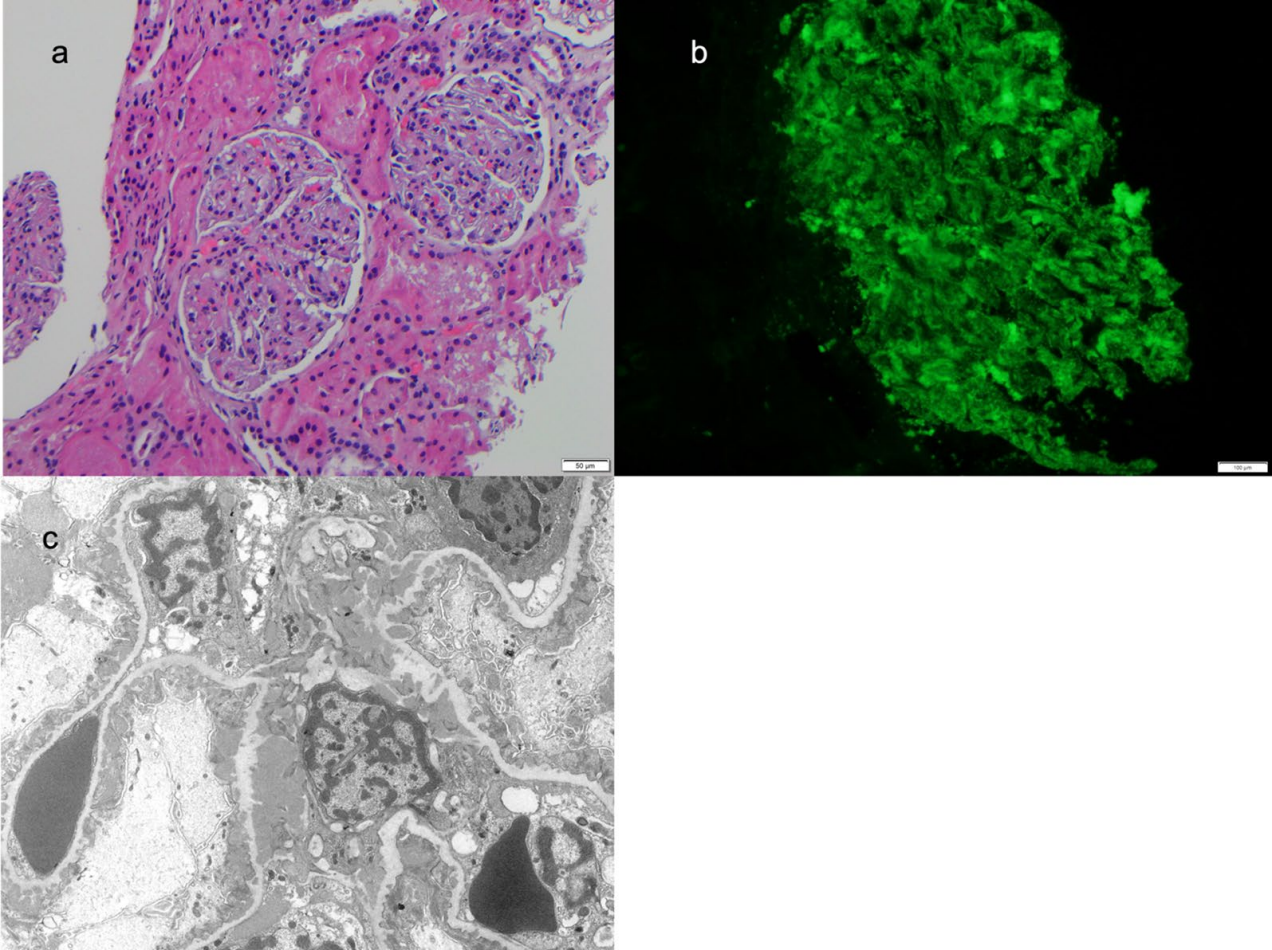
Kidney Biopsy showing **a** light microscopy, **b** immunofluorescence and **c** electron microscopy. **a** By light microscopy, glomeruli had a mild increase in mesangial matrix and cells with intact appearing basement membranes. The interstitium, tubules, and small vessels were normal except for abundant protein droplets the tubular epithelium. **b** Granular mesangial and peripheral staining was present on direct antibody immunofluorescence with antibodies against IgA, IgG, IgM, C3, and C1q. **c** Ultrastructural examination confirmed abundant mesangial and paramesangial deposits. Numerous early subepithelial membranous deposits were associated with small basement membrane spikes. Subendothelial tubuloreticular bodies were present (not shown)

The patient was started on hydroxychloroquine 300 mg daily, prednisone 60 mg daily, and famotidine 20 mg daily. He was referred to pediatric nephrology, which led to a kidney biopsy. The kidney biopsy revealed class V lupus nephritis (Fig. 1). On light microscopy, glomeruli had a mild increase in mesangial matrix and cells; the basement membranes appeared intact (Fig. 1a). The immunofluorescent evaluation demonstrated “full house” staining; he was positive for IgA, IgG, IgM, C3, and C1q (Fig. 1b). Electron microscopic examination revealed numerous subepithelial immune complex deposits with minimal basement membrane remodeling and extensive foot process effacement (Fig. 1c). Numerous deposits were noted in the mesangium along with tubuloreticular inclusions. The patient was started on mycophenolate mofetil 1000 mg twice daily and losartan 12.5 mg daily. Three weeks after initiation of prednisone, the patient had resolution of his malar rash and arthritis with improvement of his capillary changes. Additionally, his urine protein/creatinine ratio dramatically improved to 0.8 mg/mg four weeks after starting treatment.

## Discussion and conclusion

We describe a 14-year-old male patient who developed cSLE with class V lupus nephritis two days after SARS-CoV-2 mRNA Pfizer-BioNTech vaccination. This association does not prove causality. Indeed, there have been billions of COVID-19 vaccine doses given worldwide so some medical events will inevitably occur after vaccination. At the time of this case, the Centers for Disease Control was recommending a two-dose primary series for those 5–17 years of age (Pfizer and Moderna) and booster vaccine for those who had received the Pfizer vaccine [17]. However, COVID-19 vaccination has been linked with rare autoimmune-mediated adverse events, and thus it s important to be aware of potential associations given the limited data on rare adverse events with these vaccinations [18, 19].

There are a number of prior cases in adults describing an association between COVID-19 vaccination and development of SLE. Cases reporting an association of COVID-19 vaccination and the development of SLE are summarized in Table 2 [9–11, 20–27]. Overall, these cases do not demonstrate any clear pattern. Six of the 11 cases occurred in patients in their 20’s, a peak time for onset of SLE. Three different vaccines were associated with the development of SLE, and six and five cases occurred after the 2nd dose and 1st dose, respectively. The clinical manifestations were typical of SLE, but variable, with two patients having lupus nephritis. Most patients developed symptoms one to two weeks following vaccination (Table 2), in contrast with our case which occurred approximately two days after vaccination. Additionally, there have been reports of other vaccines triggering development of SLE [1–5, 13, 28, 29].

**Table 2:** Review of adult case reports associating SLE development and COVID-19 vaccination

Case report	Vaccine administered	Clinical symptoms	Family history of autoimmunity	Laboratory values/imaging/ biopsy results	Medications utilized
Baez et al. Case Rep Rheumatology Feb 2022	Moderna COVID-19 vaccine (2nd dose)	27-year-old Female with Type 1 Diabetes developed arthritis two weeks after administration	Mother with SLE	Positive antinuclear antibody, anti-dsDNA, anti-Ro, and anti-La/SSB antibodies; low C4 levels	Low dose prednisone and hydroxychloroquine
Kaur et al Cureus 2022 Feb	Pfizer-BioNTech COVID-19 vaccine (2nd dose)	54-year-old male with history of Sjögren’s syndrome; developed fever, lymphadenopathy, and purpuric lesions two weeks after vaccine	None reported	Hypocomplementemia, Positive antinuclear antibody, anti-dsDNA antibodies, anti-Smith antibodies, anti-ribonucleoprotein antibodies, anti-histone antibodies	High dose prednisone
Hidaka et al. Int J Hematol 2022 Feb	Pfizer-BioNTech COVID-19 vaccine (2nd dose)	53-year -old female with history of bronchial asthma, Vogt–Koyanagi–Harada disease, and Hashimoto disease; developed wheezing and conjunctival pallor two weeks after vaccine	None reported	Positive antinuclear antibody, hemolytic anemia, positive Coombs, thrombocytopenia, hypocomplementemia, positive lupus anticoagulant	High dose prednisone
Nune et al. Int Journal Medicine 2021	Pfizer-BioNTech SARS-CoV-2 vaccine (2nd dose)	24-year-old male developed polyarthralgia, fever and fatigue two weeks after vaccine	None reported	Positive antinuclear antibody, anti-dsDNA, lymphopenia, hypocomplementemia	High dose prednisone, methotrexate
Mousa et al. Clin Rheumatol. 2022 May	Pfizer-BioNTech COVID-19 vaccine (1st dose)	22-year-old female developed abdominal pain, vomiting, and rash one week after vaccine	No family history of autoimmunity	Positive antinuclear antibody, anti-dsDNA, lymphopenia, anemia, thrombocytopenia, transaminitis, elevated lipase and amylase, hypocomplementemia	High dose prednisone, hydroxychloroquine, azathioprine
Zavala-Miranda et al. Kidney Int. 2021 Dec	AstraZeneca CoV-19 vaccine (1st dose)	23-year-old woman who presented with nephrotic syndrome 1 week after vaccine	No family history of autoimmunity	Positive antinuclear antibody, anti-dsDNA, lymphopenia, elevated protein-to-creatinine ratio, class V Lupus Nephritis	High dose prednisone, mycophenolate mofetil, hydroxychloroquine, and diuretics
Kim et al. Kidney Int. 2022 April	AstraZeneca CoV-19 vaccine (2nd dose)	60-year-old woman with history of positive ANA developed fevers and pitting edema eight weeks after administration	None reported	Positive antinuclear antibody, anti-dsDNA, anti-Smith, lymphopenia, anemia, thrombocytopenia, elevated creatinine, elevated protein-to-creatinine ratio, class III Lupus Nephritis	Intravenous methylprednisolone, cyclophosphamide, High dose prednisone, hydroxychloroquine
Rios et al. Mod Rheumatol Case Rep. 2022 March	Pfizer/BioNTech COVID-19 vaccine (1st dose)	42-year-old woman who developed inflammatory arthritis with sudden onset dyspnea and hypoxemia 2 weeks after administration	None reported	Positive antinuclear antibody, anti-dsDNA, lymphopenia, hypocomplementemia, positive lupus anticoagulant, elevated D-dimer, CT pulmonary angiogram consistent with filling defect in the right pulmonary artery	Intravenous methylprednisolone, high dose prednisone, hydroxychloroquine, anticoagulation, azathioprine
Lemoine et al. Clin Rheumatol. 2022	Pfizer-BioNTech COVID-19 vaccine (1st dose)	68-year-old woman who presented with upper and lower extremity muscle weakness, stiffness, and pain along with subsequent rash one week after administration	None reported	Positive antinuclear antibody, anti-dsDNA	High dose prednisone, azathioprine, methotrexate
Raviv et al. Case Rep Rheumatol. 2022 Feb	Pfizer-BioNTech COVID-19 vaccine (1st dose)	24-year-old male developed facial rash 2 days after vaccine administration, followed by development of inflammatory arthritis with hair loss eight weeks after administration	No family history of autoimmunity	Positive antinuclear antibody, antichromatin antibody, ribosome P antibody, hypocomplementemia	Topical steroid cream, hydroxychloroquine etoricoxib
Patil et al. J Cosmet Dermatol. 2021 Oct	AstraZeneca CoV-19 vaccine (2nd dose)	22‐year‐old female with history of jaundice developed fever, polyarthralgia, rash, lower extremity edema and petechia one week after administration	Sister with autoimmune thyroiditis	Positive antinuclear antibody, anti-dsDNA antibody, anti-histone antibody, hemolytic anemia, positive Coombs, thrombocytopenia, elevated urine albumin 1+	High dose prednisone, hydroxychloroquine, mycophenolate mofetil, furosemide, telmisartan

*ANA* antinuclear antibody, *dsDNA* double-stranded deoxyribonucleic acid, *RNP* ribonucleoprotein, *CT* computed tomography scan

A number of studies in adults have systematically assessed SLE patient for flares following COVID-19 immunization. In one study, patients had SLE Disease Activity Index (SLEDAI) measured before and after COVID-19 vaccination (BNT162b2 [Pfizer/ BioNTech], mRNA-1273 [Moderna] or Ad26.COV2.S [Johnson & Johnson]). The SLEDAI score did not change significantly (3.2 pre and 2.9 post). There were post-vaccination flares in 11.4% of patients, but all except for one of the 11 flares were considered mild, and most did not require intervention [14].

In a prospective study of patients with rheumatic and musculoskeletal disease, including 273 patients with SLE, disease flares requiring treatment occurred in 11% of SLE patients, though none were severe [30]. Interestingly, in this study prior COVID-19 was a risk factor for a disease flare. Another study assessed disease flares in 100 patients who received the BNT162b2 (Pfizer /BioNTech) vaccine (10 only received one dose) [15]. There were 27 disease flares, more commonly after the 2nd dose. Most flares were arthritis or dermal. Importantly, there were 28 flares in the six months prior to immunization [15]. In an international cross-sectional survey of SLE patients, the flare rate was low (3%), but the severity of the flares required a change in treatment in over 70% of patients [31]. In addition, there are a number of reported cases describing flares of SLE or even transition from cutaneous SLE to systemic SLE following COVID-19 vaccination [12, 32–36].

The case reports of new onset SLE are anecdotal and are limited by their prospective, observational nature. Epidemiologic studies of immune thrombocytopenia following COVID-19 vaccine emphasize considering the baseline rate of disease before attributing a disease entity to a vaccine [19, 37]. Unfortunately, there are no current epidemiologic studies of SLE with and without preceding COVID-19 vaccination.

The pathophysiology of SLE is complex, with some individuals being at increased risk due to genetic predisposition [38]. SLE is more common in females and in people with African, Asian or Hispanic ancestry. In addition, a variety of environmental factors may have a role in initiating SLE. Ultimately, SLE is an autoimmune disease caused by disturbances in the regulation of the immune system [38].

There are potential mechanistic links between COVID-19 vaccination and SLE. The COVID-19 mRNA vaccines increase type I interferon, which is also increased and believed to be important in the pathogenesis of SLE [39]. In addition, molecular mimicry of the SARS-CoV-2 spike protein could lead to autoantibodies to self-antigens. Vaccination may also directly activate B cells. However, SLE is clearly a rare complication and may only occur in a genetically susceptible patient [40].

In summary, we have reported a pediatric patient who developed symptoms of SLE two days after his third COVID-19 mRNA vaccination. Large epidemiologic studies are needed to assess whether this is more than an association, but it would clearly be a rare complication. It is possible that the vaccination led to SLE in a genetically susceptible individual. To the best of our knowledge, this is the first reported pediatric patient with new onset SLE following COVID-19 vaccination.

## Data Availability

Data were ethically extracted from the patient’s file. Data used in this study is available from the corresponding author upon request.

## Abbreviations

SLE: Systemic lupus erythematosus
cSLE: Childhood onset SLE
SARS-CoV-2: Severe acute respiratory syndrome coronavirus 2
ANA: Antinuclear antibody
dsDNA: Double-stranded deoxyribonucleic acid
RNP: Ribonucleoprotein
ESR: Erythrocyte sedimentation rate
LN: Lupus nephritis
Ig: Immunoglobulins
